# A combination of misoprostol and estradiol for preoperative cervical ripening in postmenopausal women: a randomised controlled trial

**DOI:** 10.1111/j.1471-0528.2009.02435.x

**Published:** 2010-01

**Authors:** KS Oppegaard, M Lieng, A Berg, O Istre, E Qvigstad, B-I Nesheim

**Affiliations:** a Department of Gynaecology, Helse Finnmark, Klinikk HammerfestHammerfest, Norway; b Department of Obstetrics and Gynaecology, Oslo University Hospital UllevålOslo, Norway

**Keywords:** Cervical ripening, estradiol, hysteroscopy, misoprostol, postmenopausal, sequential trial

## Abstract

**Objective:**

To compare the impact of 1000 μg of self-administered vaginal misoprostol versus self-administered vaginal placebo on preoperative cervical ripening after 2 weeks of pretreatment with estradiol vaginal tablets in postmenopausal women prior to day-care operative hysteroscopy.

**Design:**

Randomised, double-blind, placebo-controlled sequential trial.

**Setting:**

Norwegian university teaching hospital.

**Population:**

Sixty-seven postmenopausal women referred for day-care operative hysteroscopy.

**Methods:**

The women were randomised to receive either 1000 μg of self-administered vaginal misoprostol or self-administered vaginal placebo on the evening before day-care operative hysteroscopy. All women had administered a 25-μg vaginal estradiol tablet daily for 14 days prior to the operation.

**Main outcome measures:**

Primary outcome: preoperative cervical dilatation at hysteroscopy. Secondary outcomes: difference in dilatation at recruitment and before hysteroscopy, number of women who achieved a preoperative cervical dilatation of 5 mm or more, acceptability, complications and adverse effects.

**Results:**

The mean cervical dilatation was 5.7 mm (SD, 1.6 mm) in the misoprostol group and 4.7 mm (SD, 1.5 mm) in the placebo group, the mean difference in cervical dilatation being 1.0 mm (95% CI, 0.2–1.7 mm). Self-administered vaginal misoprostol of 1000 μg at home on the evening before day-care hysteroscopy is safe and highly acceptable, although a small proportion of women experienced lower abdominal pain.

**Conclusions:**

One thousand micrograms of self-administered vaginal misoprostol, 12 hours prior to day-care hysteroscopy, after 14 days of pretreatment with vaginal estradiol, has a significant cervical ripening effect compared with placebo in postmenopausal women.

## Introduction

Ripening or softening of the uterine cervical tissue is a process involving various inflammatory reactions,^1^,^2^ although the mechanisms are poorly understood. Ripening of the cervix in early pregnancy is recommended before surgical abortion to facilitate cervical dilatation,^3^ later in pregnancy to induce labour,^4^ and in non-pregnant women if intrauterine surgery is indicated.^5^,^6^ Several methods are effective for ripening the cervix: mechanically with osmotic dilators^7^ or balloon catheters,^8^ and biochemically with prostaglandins,^9^ antiprogestins^10^ or nitric oxide donors,^11^ although prostaglandins are the most commonly used agent for cervical ripening and labour induction.

Cervical ripening and labour induction occur by the activation of the prostaglandin E2 receptor by prostaglandins.^12^ The prostaglandin E1 analogue misoprostol, originally developed as an antigastric ulcer drug,^13^ has superseded licensed prostaglandins used for cervical ripening and labour induction. It is mostly used off-label, as the patent holders who developed the drug have never applied for licences for gynaecological or obstetrical use. There is abundant literature supporting its safety and effectiveness for multiple reproductive health indications.^14^

Many patients require cervical dilatation prior to operative hysteroscopy, depending on the size of the instrument used for the operation. An operative hysteroscope/resectoscope used to treat endometrial pathology typically has a diameter of 10 mm. In women with a firmly closed and rigid cervix, dilatation can lead to considerable traumatisation of the tissue. Furthermore, complicated cervical dilatation is attended by the risk of lacerations caused by the tenaculum, the creation of false passages and an increased risk of uterine perforation. Maximum forces of more than 100 N have been measured for dilatation up to Hegar 6.^15^ Nulliparous and postmenopausal women are particularly at risk of complications.^16^–^20^

Prostaglandins have been used for cervical ripening prior to hysteroscopy since 1985.^21^ The analogues licensed for gynaecological use are both expensive and impractical to use for this purpose (e.g. intracervical application of sulprostone gel). As misoprostol is inexpensive, easy to obtain and to use, and has a long shelf-life, it has been employed as a cervical ripening agent before operative hysteroscopy in order to reduce the complications occurring during the dilatation procedure.^22^ In a previous study, we found that the cervix of over 40% of postmenopausal women was judged to be difficult to dilate and that misoprostol appeared to be effective for cervical ripening in premenopausal, but not postmenopausal, women.^23^ A relative lack of estrogen in postmenopausal women could possibly be the main reason why misoprostol appears to have no significant cervical ripening effect.

## Hypothesis

The null hypothesis is that there is no clinically significant difference in preoperative cervical dilatation just before hysteroscopy (<1 mm) between postmenopausal women who receive vaginal misoprostol and postmenopausal women who receive vaginal placebo. Both groups were pretreated with vaginal estradiol for 14 days.

## Methods

### Patients

We refer to the previously published trial protocol for comprehensive methodological details.^24^ Those eligible for the study were all postmenopausal women (defined as at least 1 year after the last menstrual period/menstruation) referred consecutively for day-care operative hysteroscopy, and who had provided informed written consent. Women were excluded if they did not want to participate, if they were medically unfit for hysteroscopy or for participation in any clinical trial, or if they did not have a medical indication for hysteroscopy. Other exclusion criteria were previous or current breast or gynaecological cancer, a medical contraindication for locally applied estradiol or misoprostol, the current use of hormone therapy or unable to communicate in Norwegian.

### Procedures

Before a cervical smear and endometrial biopsy were taken as part of the routine outpatient examination, cervical dilatation was measured by passing Hegar dilators through the cervix in ascending order, starting with a Hegar dilator of size 2 mm. Women participating in the study started daily treatment with 25-μg estradiol vaginal tablets for 2 weeks before operative hysteroscopy. Participants were randomly assigned at a ratio of 1:1 to 1000 μg of vaginal misoprostol or placebo, according to a randomised list of three permuted blocks on a double-blind basis at the outpatient consultation. The hospital pharmacist manufactured the study drug and placebo capsules, which were delivered in numbered containers. The study participants were instructed to insert the misoprostol/placebo capsules vaginally, as deep as possible, after voiding urine at approximately 21.00 hours on the evening before the operation. Both patient and examiner were blind to randomisation.

The primary efficacy outcome was the degree of cervical dilatation, immediately before hysteroscopy, assessed by passing Hegar dilators through the cervix in ascending order, starting with a size of 2 mm. Six experienced senior gynaecologists performed the operative hysteroscopies during the study period. Prior to the operation on the first woman, the project leader individually instructed the operators in how to assess preoperative cervical dilatation in order to obtain valid and reliable measurements. The size of the largest dilator passed into the internal cervical os without subjective resistance felt by the operator was recorded as the preoperative degree of dilatation. Secondary outcomes included the number of dilatations judged as ‘easy’ or ‘difficult’ by the operator, the time used to dilate the cervix, and the difference between the baseline cervical dilatation at recruitment and preoperative dilatation at hysteroscopy. Further data recorded were the symptoms and adverse effects experienced between the insertion of capsules and the operation, as well as the frequency of adverse events and complications during hysteroscopy and during the 14-day follow-up period, the acceptability of the treatment by the women and the histological result.

The trial was conducted in accordance with the Declaration of Helsinki and national as well as local regulations. The Regional Committee for Medical Research Ethics (Regional komité for medisinsk forskningsetikk, Nord-Norge, REK Nord) in Northern Norway (Ref no. 200704112-5/MRO/400) and Oslo University Hospital, Ullevål’s Personal Data Officer (Personvernansvarlig) approved the protocol. Authorisation from the Norwegian Medicines Agency (Statens Legemiddelverk, SLV) was granted (Ref. no. 07/12515-8) for the study. The trial is registered with ClinicalTrials.gov, number NCT00572819, the European Clinical Trials Database, number 2007-004083-52, and the study protocol has been published in *BJOG*.^24^ Written informed consent was obtained from all patients before randomisation.

### Statistical analysis

A two-sample sequential Wilcoxon test, developed by Skovlund and Walløe,^25^–^27^ was used in order to reduce the number of study participants needed to reach a conclusion on the primary outcome. This method has been used previously in relatively few clinical trials, but has been proven to be easy to use and robust. It is expected to reduce the number of study participants needed to reach a conclusion, compared with the number required in a corresponding fixed sample trial.^28^,^29^ The analysis is tailored to reach a conclusion on the primary outcome as soon as the difference is statistically significant, so that as few study participants as possible are enrolled. The boundaries needed for the statistical model were calculated on the basis of the preoperative variability (SD) in cervical dilatation in postmenopausal women from our previous trial, the mean cervical dilatation being 4.1 mm and SD 2.3 mm.^23^ The design of the main trial is based on a significance level of 5% and a power of 95% against a 1-mm difference in the cervical dilatation caused by misoprostol and placebo. As it seemed clinically unlikely that the use of misoprostol would cause a constriction of the cervix, a one-sided test was chosen. The trial was halted when the boundary for significance was crossed. The estimator of normal means was applied after the sequential test as the observations were judged to be normally distributed.^30^

### Role of the funding source

No pharmaceutical company was involved in this study. The funding sources had no input into the study design, collection, analysis or interpretation of the data, report preparation or in the decision to submit the paper for publication. Research grants from the Northern Norway and Eastern Norway Regional Health Authorities funded the study.

## Results

**Figure 1:**
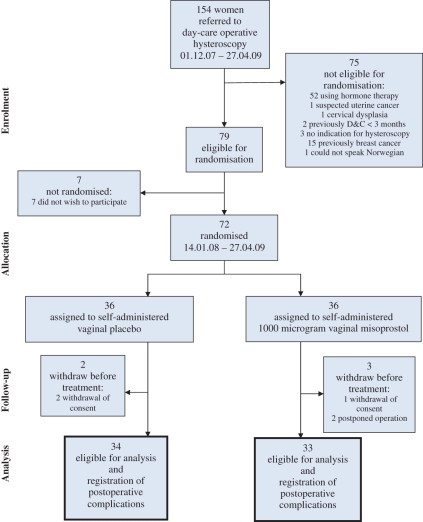
Trial profile.

**Figure 2:**
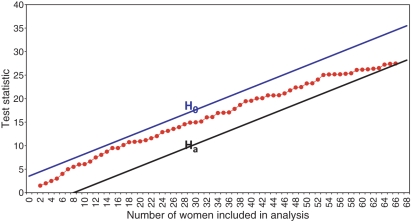
Continuation of the sequential test. The stopping boundaries and the sample path leading to the conclusion that misoprostol was significantly superior to placebo is shown. H_0_, boundary for the null hypothesis; H_a_, boundary for the alternative hypothesis.

Between January 2008 and April 2009, 72 women were enrolled and randomly assigned to treatment (Figure 1). The stopping boundaries were reached on 13 May 2009 (Figure 2) as a result of a significant difference between the misoprostol and placebo groups on the primary efficacy outcome. The groups were well balanced in terms of baseline demographics (Table 1) and surgical characteristics (Table 2). The results for the cervical dilations are shown in Table 3. The mean cervical dilatation was 5.7 mm (SD, 1.6 mm) in the misoprostol group and 4.7 mm (SD, 1.5 mm) in the placebo group, the mean difference in cervical dilatation being 1.0 mm (95% CI, 0.2–1.7 mm). The cervical dilatations were normally distributed, assessed visually with a bell curve. The main adverse effect was lower abdominal pain (Table 4), although the majority of women reported mild pain. One woman who received misoprostol reported lower abdominal pain rated at a level of six out of 10 on a visual analogue scale score, but reported that her symptoms subsided after the intake of two 500-mg paracetamol tablets. A few of the women who experienced pain reported that they had taken off-prescription analgesics (such as paracetamol and ibuprofen) with a subsequent reduction in their symptoms. No women experienced severe pain (more than six out of 10 on a visual analogue scale score). Two women in the misoprostol group (6.1%) experienced light preoperative bleeding. Of the 67 women, 47 (70%) found self-administered vaginal capsules at home to be completely acceptable, 14 (21%) fairly acceptable, five (7.5%) fairly unacceptable and one (1.5%) completely unacceptable. The main reason given for unacceptability was the lack of vaginal applicators to aid insertion. There were two similar preoperative complications, one in each treatment group: perforation of the uterine fundus with the resectoscope after dilatation. Both women were discharged on the same day as surgery and neither experienced further complications or needed further treatment. No postoperative complications were recorded during the 14-day follow-up period.

**Table 4:** Preoperative adverse effects by randomised treatment group

	Self-administered vaginal placebo (*n* = 34)	Self-administered vaginal misoprostol (*n* = 33)
No adverse effects, *n* (%)	23 (67.6)	18 (54.5)
Lower abdominal pain, *n* (%)	8 (23.5)	13 (39.4)
Mean level of reported preoperative pain* (SD)	1.0 (1.7)	1.5 (2.1)
Constipation, *n* (%)	2 (5.9)	0
Vaginal bleeding, *n* (%)	0	2 (6.1)
Vaginal discharge, *n* (%)	1 (2.9)	0

* Measured with a visual analogue scale score, ranging from 0 (no pain) to 10 (unbearable pain).

**Table 3:** Intra-operative findings, distribution of cervical dilatation and complications by randomised treatment group

	Dosage group		*P*
	Self-administered vaginal placebo (*n* = 34)	Self-administered vaginal misoprostol (*n* = 33)	
Cervical dilatation at hysteroscopy (mm), mean (SD)	4.7 (1.5)	5.7 (1.6)	0.01
Baseline cervical dilatation (mm) at inclusion, mean (SD)	2.1 (1.7)	2.6 (1.7)	
Mean difference in cervical dilatation (mm) between inclusion and hysteroscopy (SD)	2.7 (1.7)	3.1 (1.9)	
Number of patients achieving cervical dilatation ≥5 mm, *n* (%)	20 (59)	30 (88)	
‘Difficult dilatation’, *n* (%)	7 (21)	1 (3)	
Exposure to capsules (min), mean (SD)	726 (109)	750 (84)	
Frequency of preoperative complications, *n* (%)	1 (3)	1 (3)	
Complications within 14 days after hysteroscopy, *n* (%)	0	0	

**Table 2:** Indications for operative hysteroscopy and histology result by randomised treatment group

	Self-administered vaginal placebo* (*n* = 34)	Self-administered vaginal misoprostol (*n* = 33)
**Indications for hysteroscopy**		
Asymptomatic endometrial polyp or leiomyoma, *n* (%)	18 (52.9)	18 (54.5)
Postmenopausal bleeding and endometrial polyp, *n* (%)	13 (38.2)	11 (33.3)
Lower abdominal pain and endometrial polyp, *n* (%)	2 (5.9)	2 (6.1)
Vaginal discharge and endometrial polyp, *n* (%)	–	2 (6.1)
Removal of intrauterine device, *n* (%)	1 (2.9)	–
**Histology results**		
Benign histology, *n* (%)	28 (82.4)	27 (81.8)
Complex endometrial hyperplasia with atypia, *n* (%)	2 (5.9)	3 (9.1)
Complex endometrial hyperplasia without atypia, *n* (%)	1 (2.9)	1 (3.0)
Simple endometrial hyperplasia, *n* (%)	1 (2.9)	1 (3.0)
Endometrial adenocarcinoma, *n* (%)	1 (2.9)	1 (3.0)

**Table 1:** Baseline demographic characteristics of women by randomised treatment group

	Self-administered vaginal placebo (*n* = 34)	Self-administered vaginal misoprostol (*n* = 33)
Age (years), mean (SD)	62.4 (6.0)	60.5 (6.8)
Body mass index (kg/m^2^), mean (SD)	26.6 (5.1)	28.4 (5.4)
Years of menopause, mean (SD)	12.1 (6.1)	9.9 (6.8)
Total number of children born, mean (SD)	2.0 (1.0)	2.1 (1.2)
Number of vaginal deliveries, mean (SD)	1.9 (1.1)	2.0 (1.2)
Women delivered with caesarean sections, *n* (%)	2 (5.9)	2 (6.1)
Women with previous cervical dilatation, *n* (%)	16 (47.1)	14 (42.4)
Women with previous cone biopsy, *n* (%)	2 (5.9)	2 (6.1)

## Discussion

Our trial shows that 1000 μg of misoprostol self-administered vaginally 12 hours before day-care hysteroscopy is safe and effective for cervical ripening compared with placebo in postmenopausal women after the use of vaginal estradiol tablets daily for 14 days. This regimen is highly acceptable and easy to use. Adverse effects were few and comparatively minor, the main one being lower abdominal pain (the maximum experienced being described as ‘moderate’) after taking the capsules. The Norwegian Medicines Agency required us to inform all study participants of the risk of lower abdominal pain as a possible adverse effect, which may explain why 24% of women in the placebo group reported lower abdominal pain after taking the capsules.

The main strength of this study was its design to demonstrate whether the vaginal administration of misoprostol after pretreatment with estradiol could result in a significant difference in cervical dilatation compared with placebo in postmenopausal women with the smallest possible number of study participants. It is an important ethical issue to reach this conclusion with as few study subjects as possible exposed to an ineffective drug. The study was designed and conducted strictly in adherence with the CONSORT criteria,^31^ and the protocol was submitted for peer review before the trial started to recruit women. Furthermore, the study included all consecutive women referred to outpatient hysteroscopy. This treatment is also inexpensive: misoprostol is a stable drug with a long shelf-life and does not need to be kept refrigerated. Self-administration does not require resources from staff — apart from information — and is not time-consuming. Five 200-μg tablets of misoprostol (Cytotec®, Pfizer, Morpeth, UK) cost less than two euros in Norway, and a packet of 15, 25-μg estradiol tablets (Vagifem®, Novo Nordisk, Måløv, Denmark) costs 16 euros. In addition to these costs, the cost of standard, off-prescription analgesics adds little to the total therapeutic cost prior to operative hysteroscopy. It is difficult to calculate a cost–benefit advantage when using ‘complications avoided’ as an endpoint, but there were fewer complications and fewer difficult dilatations in this study when compared with the results in a previous investigation.^23^

The main weakness in this study was the validity of evaluation of the primary efficacy outcome by each gynaecologist. The use of preoperative cervical dilatation as a primary outcome for the assessment of cervical ripening is open to discussion. However, it is a commonly used outcome in randomised trials on this topic.^9^,^22^ The use of other methods, such as a tonometer, to judge the resistance to cervical dilatation might have provided more objective results, but this is not a routine procedure in connection with hysteroscopy. Outcomes such as ‘difficulty in dilating the cervix’ and ‘pain experienced by the women’ might have been open to even greater subjectivity and a much wider variation in results. A secondary outcome measure of cervical dilatation of 5 mm was used to reinforce the impression of the cervical ripening effect caused by misoprostol. Miniature hysteroscopes do not necessarily require cervical dilatation, but a 10-mm resectoscope almost always does. A 1-mm difference in cervical dilatation might be a questionable outcome if there were only a few women in the comparison. Used in a trial with a sufficient number of trial participants, this might be a statistically and clinically significant difference. If no difference between the groups was found with an outcome measure of 1 mm and a sufficiently high power, one could be reasonably sure that misoprostol had no effect. In retrospect, we conclude that we should not have included women who had previously undergone cervical surgery as eligible for our study. The postmenopausal women who had previously undergone cone biopsy in our study had a substantial portion of their cervix removed, and neither misoprostol nor estradiol had any ripening effect on the remaining tissue. The study took longer to complete than expected because of the unexpectedly high number of women referred using hormone replacement therapy independent of the trial, and who therefore were consequently not eligible for inclusion.

There have been few published studies on the effect of misoprostol on the postmenopausal cervix, and the results are inconclusive. Three studies did not find a significant ripening effect of misoprostol compared with placebo.^15^,^32^,^33^ Two studies have suggested that misoprostol is effective for cervical ripening in postmenopausal women,^34^,^35^ but both studies included postmenopausal women as a subgroup in their analysis. None of these studies recorded whether the women were using hormone replacement therapy.

We found only one previous study that investigated the ripening effect of misoprostol on the estrogen-pretreated cervix in postmenopausal women.^36^ The authors concluded that misoprostol alone was ineffective for cervical ripening, but that it was effective in 22 women after they had used estriol vaginal cream for 14 days prior to dilatation.

Although we could not find any literature linking misoprostol’s cervical ripening effect to the presence or absence of estrogen, we believe that there is evidence to suggest a connection. Estrogen appears to be essential for cervical ripening to take place.^37^ Pregnant women with placental sulphatase deficiency (resulting in low circulating estrogens) do not show ripening of their cervix.^38^–^42^ The inflammatory cascade during the cervical ripening process involves leucocytes, and the presence of estrogen receptors on cervical leucocytes^43^ suggests that estrogen may directly regulate leucocyte function in the cervix.^44^ The local application of estrogen for the induction of labour has been tried,^45^ and estrogen does enhance cervical ripening.^46^ However, estrogens appear to be less effective than prostaglandins for the induction of labour and delivery, and there are insufficient data to draw any conclusions.^47^ The effect of estrogen alone on cervical ripening in postmenopausal women has not been investigated previously. We noticed that there was a remarkable difference in cervical dilatation in all women between the initial measurement taken at the outpatient’s consultation and that taken just before hysteroscopy. The postmenopausal women in the placebo group were also noticeably easier to dilate compared with the postmenopausal women receiving placebo in our previous study (who had not used hormone therapy). This suggests the possibility that estradiol alone has a substantial effect on the remodelling and ripening of cervical tissue in only 14 days. Further studies are needed to confirm this, as our trial was only designed to measure the difference in cervical dilatation between the misoprostol and placebo groups. Future trials investigating the ripening effect of misoprostol on the postmenopausal cervix must also include information on whether women are using hormone therapy. We suspect that the varying effect of misoprostol on cervical ripening may have been caused by different levels of circulating estrogens. Women who are in early pregnancy require much higher doses of misoprostol for cervical ripening, compared with women at term.^14^ Twenty-five micrograms of misoprostol are sufficient to initiate cervical ripening and to induce labour in pregnant women at term, whereas doses of 800 μg are recommended for the induction of first-trimester abortion. We also found misoprostol to be much less effective in non-pregnant women than in pregnant women in our previous studies.^23^,^48^,^49^

Misoprostol is a safe and well-tolerated drug. The dosages chosen for previous trials on non-pregnant women have been similar to those used prior to the termination of pregnancy (the majority using 400 μg of misoprostol). Misoprostol generally has a far weaker and more unpredictable cervical ripening effect in non-pregnant (pre- and postmenopausal) than pregnant women. This could explain why other previous trials have concluded that misoprostol is ineffective for cervical ripening with lower doses and other administration methods, compared with placebo. The only previous trial we found that described the use of 1000 μg of vaginal misoprostol for cervical ripening concluded that it was not effective if given 2–4 hours before surgery.^50^ We chose a higher dosage of vaginally administered misoprostol, and a longer administration to operation interval, because there was no clear consensus from previous trials as to whether misoprostol has a significant ripening effect in non-pregnant women. Such a large dose given orally would probably result in significant gastrointestinal and possibly other systemic adverse effects. The failure of misoprostol in previous trials to have any effect on cervical ripening may have been the result of an inadequate dosage or insufficient time between the delivery of the tablets and operation.

Long-term estradiol therapy is contraindicated in women who have previously had, or have current or suspected, breast cancer, current or suspected estrogen-dependent tumours, untreated endometrial hyperplasia, undiagnosed genital bleeding, deep-vein thrombosis or porphyria. As far as we are aware, there is no evidence to suggest that 14 days of local 25-μg estradiol treatment would pose a safety risk to women, although every woman must be assessed before treatment is prescribed on an individual basis.

## Conclusions

One thousand micrograms of self-administered vaginal misoprostol, administered 12 hours prior to operative hysteroscopy, has a significant cervical ripening effect compared with placebo in postmenopausal women after 14 days of pretreatment with vaginal estradiol. Estrogen therapy appears to be necessary for cervical ripening to occur with misoprostol in postmenopausal women. Self-administered vaginal administration of 1000 μg of misoprostol at home on the evening before operative hysteroscopy is safe and highly acceptable, although there is a risk of lower abdominal pain and light preoperative bleeding with this regimen, and women should be made aware of this and offered standard analgesics. Based on the results of our study, we believe the practical dose and timing in postmenopausal women to be 1000 μg of misoprostol self-administered vaginally at home about 12 hours before operative hysteroscopy, after 14 days of pretreatment with vaginal estradiol. We would recommend offering this inexpensive and easy to use regimen to postmenopausal women prior to undergoing operative hysteroscopy to reduce the risk of complications and to facilitate cervical dilatation.
